# The prognostic role of C-KIT, TET1 and TET2 gene expression in Acute Myeloid Leukemia

**DOI:** 10.1007/s11033-022-08000-0

**Published:** 2022-11-12

**Authors:** Reem Nabil, Naglaa M. Hassan, Mona S. Abdellateif, Rania M. Gawdat, Samar Sami Elshazly

**Affiliations:** 1 Clinical pathology Department, National Cancer Institute, Cairo University, Giza, Egypt; 2 Medical Biochemistry and molecular biology, Cancer Biology Department, National Cancer Institute, Cairo University, Giza, Egypt; 3 Clinical and chemical pathology department, Faculty of medicine, Beni Suef university, Beni Suef, Egypt

**Keywords:** AML, *C-KIT*, *TET1*, *TET2*

## Abstract

**Aim:**

was to assess the role of *C-KIT*, *TET1* and *TET2* expression in the diagnosis and prognosis of acute myeloblastic leukemia (AML).

**Methods:**

The expression levels of *C-KIT, TET1* and *TET2* were assessed in the bone marrow (BM) aspirate of 152 AML patients compared to 20 healthy control using quantitative real-time polymerase chain reaction (qRT-PCR). Data were correlated with the clinico-pathological features of the patients, response to treatment, disease-free survival (DFS), and overall survival (OS) rates.

**Results:**

*C-KIT, TET1* and *TET2* were significantly upregulated in AML patients [0.25 (0–11.6), 0.0113 (0–3.301), and 0.07 (0–4); respectively], compared to the control group [0.013 (0.005–0.250), P < 0.001, 0.001 (0–0.006), P < 0.001, and 0.02 (0.008–0.055), P = 0.019; respectively]. The sensitivity, specificity, and area under curve of of *C-KIT* were (48.7%, 100%, 0.855; respectively, P = 0.001), and that of *TET1* were (63.4%, 100%, 0.897; respectively, P = 0.001), while that of *TET2* were (56.8%, 100%, 0.766; respectively, P = 0.019). When combining the three markers, the sensitivity was 77.5%, however it reached the highest sensitivity (78.6%) and specificity (100%) when combining both *c-KIT + TET1* together for the diagnosis of AML. *C-KIT* overexpression associated with shorter DFS (P = 0.05) and increased incidence of relapse (P = 0.019). Lymph nodes involvement [HR = 2.200, P = 0.005] is an independent risk factor for shorter OS rate of AML patients. Increased BM blast % [HR = 7.768, P = 0.002], and *FLT3-ITD* mutation [HR = 2.989, P = 0.032] are independent risk factors for shorter DSF rate of the patients.

**Conclusion:**

*C-KIT, TET1*, and *TET2* could be used as possible useful biomarkers for the diagnosis of AML.

## Introduction

Leukemias are widespread hematological malignancies that are characterized by an uncontrolled proliferation of hematopoietic precursor blast cells, that interfere with the normal maturation and functions of the leukocytes [1]. Acute myeloid leukemia (AML) is the most common leukemia that occurs in adult and pediatric patients, which is characterized by an increased incidence of relapse and poor outcomes for the patients [2]. It is a heterogeneous disease with a multistep process [3]. Hence, the recent risk stratification of the AML, e.g. The World Health Organization (WHO) [4] and European LeukemiaNet (ELN) classifications [5] depends upon the underlying genetic mutations together with the associated cytogenetic abnormalities to guide the therapeutic plan, and therefore, it will help in predicting the prognosis, outcomes, and response to treatment [5, 6]. The included genetic mutations which are widely used in the clinical practice for the risk stratification of the patients are tumor protein 53 (*TP53*), internal tandem duplication of FMS-like tyrosine kinase 3 (*FLT3-ITD*), *CCAAT*/enhancer-binding protein alpha (*CEBPA*), and nucleophosmin 1 (*NPM1*) [5].

The *C-KIT* oncogene encodes a class III transmembrane receptor tyrosine kinase ( CD117) [7]. It is expressed in less than 5% of marrow myeloid progenitor cells [8], and it has an important role in the processes of cell proliferation, survival, adhesion, and chemotaxis [9]. Also, C-KIT expression is found in the blasts of 70% of the AML patients [10]. It had been reported that the overexpression of both wild and mutant types *C-KIT* can promote leukemogenesis [7, 11]. While its down-regulation can inhibit the activity of the hematological malignant cells [12].

Recently, there are many epigenetic mutations have been founded to have a role in the pathogenesis and development of AML. These epigenetic regulators included the family of ten-eleven translocation (*TET*) proteins, which facilitate DNA demethylation [13]. The tet oncogene family is composed of three isoenzymes, *TET1, TET2*, and TET3, which convert 5-methylcytosine (5mC) to 5-hydroxymethylcytosine (5hmC), formylcytosine (5fC), and carboxylcytosine (5caC) in a stepwise manner [14], which leads to demethylation, gene activation and consequently cellular proliferation [15]. There were some previous studies illusterated the significant relation between *TET1* and T*ET2* gene expression with AML prognosis [16, 17].

TET1 has been found as a fusion partner of the MLL gene associated with t (10;11)(q22;q23) in AML [18, 19]. It plays an oncogenic role in AML as it was found that it promots expression of oncogenic targets (e.g., HOXA9, MEIS1, PBX3, etc.) and represses the expression of tumor suppressor targets (e.g., miR-22) [20, 21]. TET1 was significantly upregulated in MLL-rearranged AML [20–22]. Moreover Zhao et al. confirmed the essential oncogenic role of TET1 in the development of myeloid malignancies [15], Wang et al., found high TET1 expressers harbored poor overall survival in CN-AML patients [17]. For that, targeting TET1 signaling is a promising therapeutic strategy to treat TET1high AML patients [23]. Jiang et al., identified two compounds (i.e., NSC-311,068 and NSC-370,284) that selectively suppress TET1 transcription and 5hydroxymethylcytosine (5hmC) modification, that effectively inhibit cell viability in AML with high expression of TET1, including AML with MLL rearrangements and t(8;21). Also, they demonstrated that NSC-311,068 and especially NSC-370,284 significantly repressed TET1-high AML progression in vivo [23].

TET2 has a role in haematopoiesis as it reversed aberrant hematopoietic stem and progenitor cell self-renewal in vitro and in vivo. Therefore, it suppressed human leukemic colony formation and leukemia progression of primary human leukemia patient derived xenografts [24]. Rasmussen et al., indicated that loss of TET2 in hematopoietic cells leads to DNA hypermethylation of active enhancers and induction of leukemogenesis [25]. Also TET2 mutations frequently occur in AML [26, 27]. Moreover, TET2 mutations were important prognostic factors in AML and predicted response to hypomethylating agents in Myelodysplastic syndromes (MDS) patients [28].

The expression pattern and clinical significance of TET3 have been determined in several human cancers, which indicated that it plays a different role between cancer occurrence and development [29]. Some studies found that TET3 was highly expressed in renal cell carcinoma and endometrial cancers. Also, it had been proposed that a high mRNA level of TET3 was an independent predictor of poor outcome in patients with renal cell carcinoma [30, 31]. Zhang et al., observed that TET3 expression was increased in AML patients and acted as an independent prognostic factor, that could be overcame by hematopoietic stem cell transplantation (HSCT) [29]. Whereas, several other investigations reported that TET3 was low-expressed in diverse human cancers as in cervical cancer [32], chronic lymphocytic leukemia cells [33] and in colorectal cancer [34].

Therefore, the aim of the current study is to investigate the diagnostic and prognostic values of *C-KIT, TET1*, and *TET2* expression in AML patients. This may help to clearly understand their role in AML carcinogenesis, which allows for better risk stratification of the patients to guide individualized treatment protocol. This will be achieved by assessing the association between *C-KIT, TET1*, and *TET2* expression with the patients’ clinicopathological features, response to treatment, and survival rates of the patients.

## Methods

This retrospective cohort study included 152 newly diagnosed AML patients, who were presented to the medical oncology clinics, National Cancer Institute (NCI), Cairo University, during the period from August 2016 to December 2018. Patients were compared to 20 healthy control subjects who were presented to the bone marrow transplantation unit.

Diagnosis of AML patients was performed through detailed history taking, full clinical examination, and laboratory investigations including complete blood count (CBC), BM aspiration, Immunophenotyping (IPT) of the blast cells, as well as conventional karyotyping.

The patients were classified according to the genetic risk (combined cytogenetic and molecular analysis according to Dohner et al., 2010 into three risk groups; favorable prognosis, intermediate risk group and poor prognosis [35]. Also, patients were classified according to ELN recommendations (which primarily relayed on concurrent gene mutations rather than genetic abnormalities) into Favorable risk group, intermediate risk group and adverse risk group [5].

## Treatment and follow up of the patients

The induction chemotherapy for the AML patients was formed of a combination of cytosine arabinoside (100 mg/m2) for 7 days and Adriamycin (45 mg/m2) for 3 days. Patients who achieved complete remission (CR) and had favorable cytogenetics [inv16 and t(8; 21)] received consolidation chemotherapy with a high dose of Ara-C (3gm/m2 IV infusion over 3 h/12 hours for 3 days) for a total of 3–4 cycles. Patients with high-risk cytogenetics (monosomy 7 or 5, deletion of 5q, abnormalities of 3q, and those with a complex karyotype) or intermediate-risk cytogenetics (those with normal cytogenetics and other changes not associated with high risk or favorable groups) transferred for allogeneic BM transplantation (if they had matched sibling donor) after achieving CR. Patients who did not have a matched donor received consolidation chemotherapy as in the favorable group. Patients who relapsed after conventional chemotherapy or failed to achieve CR despite optimal induction treatment received the second induction and then transferred for allogeneic BM transplantation (if they had a matched sibling donor). Patients who relapsed after bone marrow transplantation received palliative chemotherapy (High dose ARA-C, Mitoxantrone).

Follow-up of the patients was done by the clinical and BM examination on day14 and day 28 of induction treatment. The outcome of the induction treatment was assessed at D28, where patients were categorized according to their response into patients who achieved complete remission (CR), and patients who were refractory to therapy. The CR was defined in accordance with standard criteria by Dohner et al. [35], which required an absolute neutrophil count of 1.5 × 10^9^/L, platelet count of 100 × 10^9^/L or more, no blasts in the peripheral blood (PB), BM cellularity more than 20%, no Auer rods, less than 5% BM blasts and no extramedullary leukemia. Disease free survival (DFS) was calculated from the date of CR to the date of relapse or death from any cause. The overall survival (OS) was calculated from the date of diagnosis until the date of death or last follow-up.

### Sample collection

BM samples were obtained from all patients and control subjects according to the standard protocols for detection of *c-KIT, TET1*, and *TET2* gene expression levels using quantitative RT-PCR.

### Purification of total cellular RNA and cDNA synthesis

Total RNA was extracted from the BM aspirate of all subjects using QIAamp RNA blood Mini Kit (Qiagen, LOT no. 154,013,334) according to the manufacturer’s instruction. Quantitation and purity assessment of the isolated RNA were done using the Nano Drop® (ND)-1000 spectrophotometer (Nano Drop Technologies, Inc. Wilmington, USA). Reverse transcription of the RNA to cDNA was done by using the Applied Biosystems™ High-capacity cDNA Reverse Transcription Kit (Thermo Fisher Scientific, LOT no. 00716544).

### **Real-time quantification PCR of*c-Kit, TET1*and*TET2***

The RT-PCR was carried out using fluorescent TaqMan Gene Expression Assays (*c-Kit*: Hs00174029, *TET1*: Hs04189344_g1; *TET2*: Hs00325999_m1, and β-Actin as a reference gene, Thermo Fisher Scientific). The RT- PCR amplification was performed using the computerized thermocyclers (ABI step one Applied Biosystems). The expression of the target genes was determined by relative fold change in gene expression, that was calculated using the delta-delta Ct method. It was normalized to the endogenous reference gene (β-Actin) and relative to the healthy controls [36].

## Statistical analysis

Statistical analysis was done using SPSS© Statistics version 22 (IBM© Corp., Armonk, NY, USA). Data were presented as median and interquartile range (IQR) according to the performed normality tests. Qualitative data were expressed as frequency and percentage. The Area under the receiver operating curve (ROC) was calculated to investigate the best cut-off value, sensitivity and specificity for the diagnosis of AML. The relation between qualitative variables was performed using Chi-square or Fisher’s exact test as appropriate. Comparison between groups was done using the Mann-Whitney test. Survival analysis was done using Kaplan-Meier analysis, and the comparison between survival curves was done using the log-rank test. All tests were two-tailed. A p-value < 0.05 was considered significant.

## Results

The median age of the assessed AML patients was 33 (range 1–64), 35(23%) of them were lower than 18 years old, and 117 (77%) were adults. Males represented 53.3% (81/152), and females represented 46.7% (71/152). Bone marrow (BM) was hypercellular in 120 (79.5%) patients, normocellular in 27 (17.9%) patients, and hypocellular in only 4 (2.6%) patients. The most common FAB subtype was M2 in 51 (33.6%) patients, followed by M4 in 45 (29.6%) patients, and M1 in 28 (18.4%) patients. The IPT of the samples revealed that 63.3% (93/147) were myelocytic, 30.6% (45/147) were myelomonocytic, 4.1% (6/147) were monocytic, and 2.0% (3/147) were megakaryoblast. Twenty-five (16.7%) patients had mutant FLT3-ITD, 14 (23.3%) had mutant FLT3-TKD, and 24 (20.7%) had mutant NPM. Cytogenetics was favorable in 78 (58.2%) patients and adverse in 56 (41.8%) patients. There were 26 (17.1%) patients with high-risk diseases, 48 (31.6%) with intermediate-risk, and 78 (51.3%) patients with low-risk diseases. At the end of the study, relapse was reported in 18 (15.9%) patients, 54 (35.5%) patients died before day-28 of treatment (early death), and 42 (27.6%) died after day-28 (late death). Other clinical features of the patients were illustrated in Table 1.

**Table 1:** Association between *c-KIT* expression and the clinical features of the AML patients

**Parameter**		**Frequency (%)**	***c-KIT***		**P value**
			**Low expression**	**Overexpression**	
**Age (years)**	Median (IQR)	33 (1–64)	33 (1–64)	32 (1–64)	0.538
**TLC**	Median (IQR)	24.9 (1-616)	25 (1-395)	24.9 (1-616)	0.896
**Hb**	Median (IQR)	7.8 (4–14)	7.4 (4–13)	7.95 (4–14)	0.694
**PLT**	Median (IQR)	33 (4-826)	31 (6-191)	34.5 (5-283)	0.412
**PB. Blasts**	Median (IQR)	43 (0–98)	40 (0–95)	49 (0–98)	0.036
**BM blasts**	Median (IQR)	70 (20–97)	66.5 (20–97)	70 (20–97)	0.201
**gender**	male	81 (53.3%)	45 (57.7%)	36 (48.6%)	0.329
	female	71 (46.7%)	33 (42.3%)	38 (51.4%)	
**BM Cellularity Initial**	hypercellularity	120 (79.5%)	57 (73.1%)	63 (86.3%)	0.126
	normocelluarity	27 (17.9%)	18 (23.1%)	9 (12.3%)	
	hypocellularity	4 (2.6%)	3 (3.8%)	1 (1.4%)	
**FAB**	M0	3 (2.0%)	2 (2.6%)	1 (1.4%)	0.633
	M1	28 (18.4%)	12 (15.4%)	16 (21.6%)	
	M2	51 (33.6%)	25 (32.1%)	26 (35.1%)	
	M3	14 (9.2%)	9 (11.5%)	5 (6.8%)	
	M4	45 (29.6%)	23 (29.5%)	22 (29.7%)	
	M5	8 (5.3%)	6 (7.7%)	2 (2.7%)	
	M7	3 (2.0%)	1 (1.3%)	2 (2.7%)	
**IPT**	monocytic	6 (4.1%)	5 (6.6%)	1 (1.4%)	0.385
	myelocytic	93 (63.3%)	46 (60.5%)	47 (66.2%)	
	myelomonocytic	45 (30.6%)	24 (31.6%)	21 (29.6%)	
	megakaryoblastic	3 (2.0%)	1 (1.3%)	2 (2.8%)	
**FLT3-ITD**	wild	125 (83.3%)	62 (81.6%)	63 (85.1%)	0.663
	mutant	25 (16.7%)	14 (18.4%)	11 (14.9%)	
**FLT3-TKD**	wild	46 (76.7%)	24 (96.0%)	22 (62.9%)	0.004
	mutant	14 (23.3%)	1 (4.0%)	13 (37.1%)	
**NPM**	wild	92 (79.3%)	47 (82.5%)	45 (76.3%)	0.494
	mutant	24 (20.7%)	10 (17.5%)	14 (23.7%)	
**Chromosomal translocations**	negative	105 (69.5%)	57 (74.0%)	48 (64.9%)	0.385
	t(8;21)	25 (16.6%)	10 (13.0%)	15 (20.3%)	
	inv. 16	9 (6.0%)	3 (3.9%)	6 (8.1%)	
	PML/RARa	12 (7.9%)	7 (9.1%)	5 (6.8%)	
**Cytogenetics**	favorable	78 (58.2%)	34 (49.3%)	44 (67.7%)	0.036
	adverse	56 (41.8%)	35 (50.7%)	21 (32.3%)	
**genetic risk**	HR	26 (17.1%)	18 (23.1%)	8 (10.8%)	0.042
	IR	48 (31.6%)	27 (34.6%)	21 (28.4%)	
	LR	78 (51.3%)	33 (42.3%)	45 (60.8%)	
**ELN classification**	AR	13 (8.6%)	8 (10.3%)	5 (6.8%)	0.382
	IR	95 (62.5%)	51 (65.4%)	44 (59.5%)	
	FR	44 (28.9%)	19 (24.4%)	25 (33.8%)	
**Organomegaly**	no	60 (53.6%)	28 (47.5%)	32 (60.4%)	0.105
	hepatomegaly	11 (9.8%)	8 (13.6%)	3 (5.7%)	
	splenomegaly	13 (11.6%)	10 (16.9%)	3 (5.7%)	
	HSM	28 (25.0%)	13 (22.0%)	15 (28.3%)	
**LNs involvement**	no	55 (47.4%)	27 (50.0%)	28 (45.2%)	0.710
	yes	61 (52.6%)	27 (50.0%)	34 (54.8%)	
**Relapse**	no	95 (84.1%)	57 (91.9%)	38 (74.5%)	0.019
	yes	18 (15.9%)	5 (8.1%)	13 (25.5%)	
**Early death** (before D28)	no	98 (64.5%)	49 (62.8%)	49 (66.2%)	0.735
	yes	54 (35.5%)	29 (37.2%)	25 (33.8%)	
**late death** (after D28)	no	110 (72.4%)	59 (75.6%)	51 (68.9%)	0.371
	yes	42 (27.6%)	19 (24.4%)	23 (31.1%)	
**Overall Death**	no	56 (36.8%)	31 (39.7%)	25 (33.8%)	0.503
	yes	96 (63.2%)	47 (60.3%)	49 (66.2%)	

BM: bone marrow, HB: haemoglobin: IPT: immunophenotyping, PB: peripheral blood, BM: bone marrow, PLT: platelets, TLC: total leukocyte count

### Expression levels of *c-Kit, TET1* and *TET2* in AML patients

**Fig. 1:**
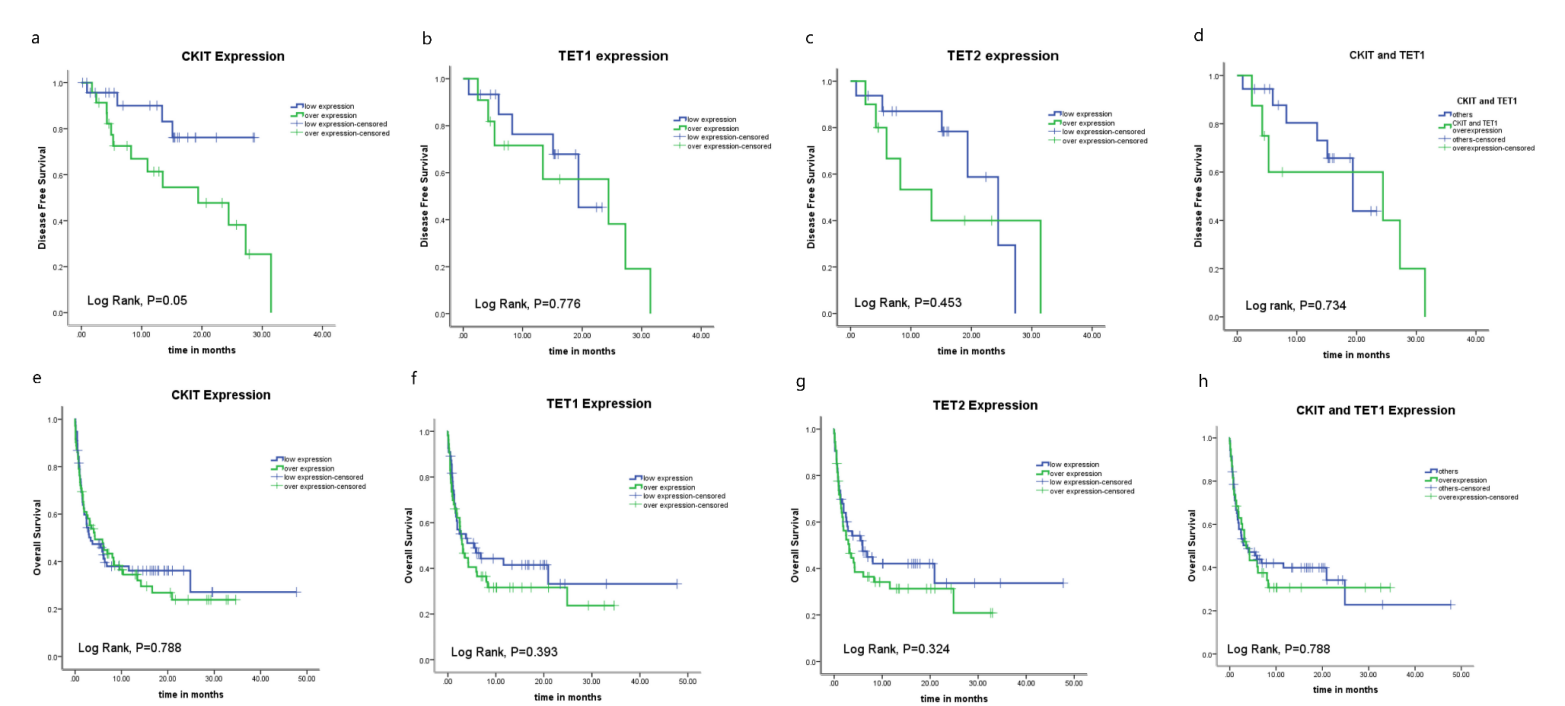
Expression levels of: (A) C-KIT, (B) TET1, and (C) TET2 in BM sample of adult AML patients compared to the normal controls. ROC analysis of (D) C-KIT, (E) TET1, and (F) TET2 for the diagnosis of AML patients compared to the normal controls

There was a significant upregulation of *c-Kit* expression level in AML patients compared to the control group [0.25 (0-11.6) and 0.013 (0.005–0.250); respectively, P < 0.001]. Similarly, *TET1* and *TET2* genes were upregulated in AML patients [0.0113 (0-3.301) and 0.07 (0–4); respectively] in comparison to the control group [0.001 (0-0.006) and 0.02 (0.008–0.055); respectively], where the significance levels were (P < 0.001 and 0.019; respectively, Fig. 1 A, B, C).

### The diagnostic value of *c-Kit, TET1* and *TET2* in AML patients

The ROC was performed to evaluate the role of *c-Kit, TET1* and *TET2* expression in the diagnosis of AML patients in comparison to the control subjects. The sensitivity of each gene was assessed at a specificity of 100% to ensure better diagnosis of the patients without the risk of false negative results.

At a specificity of 100%, the sensitivity and area under curve (AUC) of *c-Kit* were (48.7% and 0.855; respectively, with a cut-off 0.25, P < 0.001), the sensitivity and AUC of *TET1* were 63.4% and 0.897; respectively, with a cut-off 0.006, (P < 0.001), and that of *TET2* were (56.8% and 0.766; respectively, with a cut-off 0.06,P = 0.019). When combining the three markers together, the sensitivity increased to be 66.7% for *TET1 + TET2*, 77.5% for *c-KIT + TET1 + TET2*, while it was 78.6% for *c-KIT + TET1* (P < 0.001 for all, Fig. 1D, E, F; Table 2).

**Table 2:** Roc curve analysis of *c-KIT, TET1* and *TET2* for diagnosis of AML patients

	**AUC**	**Cut off**	**sensitivity**	**specificity**	**P value**	95% CI	
						**Lower Bound**	**Upper Bound**
***c-KIT***	0.855	0.25	48.7%	100%	P < 0.001	0.767	0.943
***TET 1***	0.897	0.006	63.4%	100%	P < 0.001	0.803	0.990
***TET 2***	0.766	0.06	56.8%	100%	0.019	0.651	0.880
***TET1 + TET2***	0.894	-	66.7%	100%	P < 0.001	0.804	0.984
***c-KIT + TET1***	0.915	-	78.6%	100%	P < 0.001	0.849	0.980
***c-KIT TET1 + TET2***	0.910	-	77.5%	100%	P < 0.001	0.845	0.975

AUC: area under curve, CI: confidence interval

Patients were categorized into lowexpressors and overexpressors according to the cut-off obtained by the ROC analysis for *c-Kit, TET1*, and *TET2* expression (0.25, 0.006, and 0.06; respectively).

### Univariate and multivariate logistic regression analysis of *CKIT, TET1* and *TET2* for the diagnosis of AML

The univariate logistic regression analysis showed that *CKIT, TET1*, *TET2* or *CKIT + TET1* associated significantly with AML. While the multivariate logistic regression analysis showed that only *CKIT* (OR:3.942, P = 0.012) and *TET1* (OR:4.199, P = 0.021) were considered as independent possible predictors for the diagnosis of AML patients (Table 3).

**Table 3:** Univariate and multivariate logistic regression analysis of *CKIT, TET1* and *TET2* for the diagnosis of AML

	**Univariate analysis**			**Multivariate analysis**		
	**OR**	**SE**	**P value**	**OR**	**SE**	**P value**
***CKIT***	7.091	0.322	P < 0.001	3.942	0.547	0.012
***TET1***	8.000	0.401	P < 0.001	4.199	0.620	0.021
***TET2***	7.571	0.402	P < 0.001	0.713	0.714	0.635
***CKIT + TET1***	10.286	0.396	P < 0.001	17.9	0.427	0.997

OR: odds ratio, SE: standard error

### Association between *c-KIT* expression and the clinical features of the patients

**Fig. 2:**
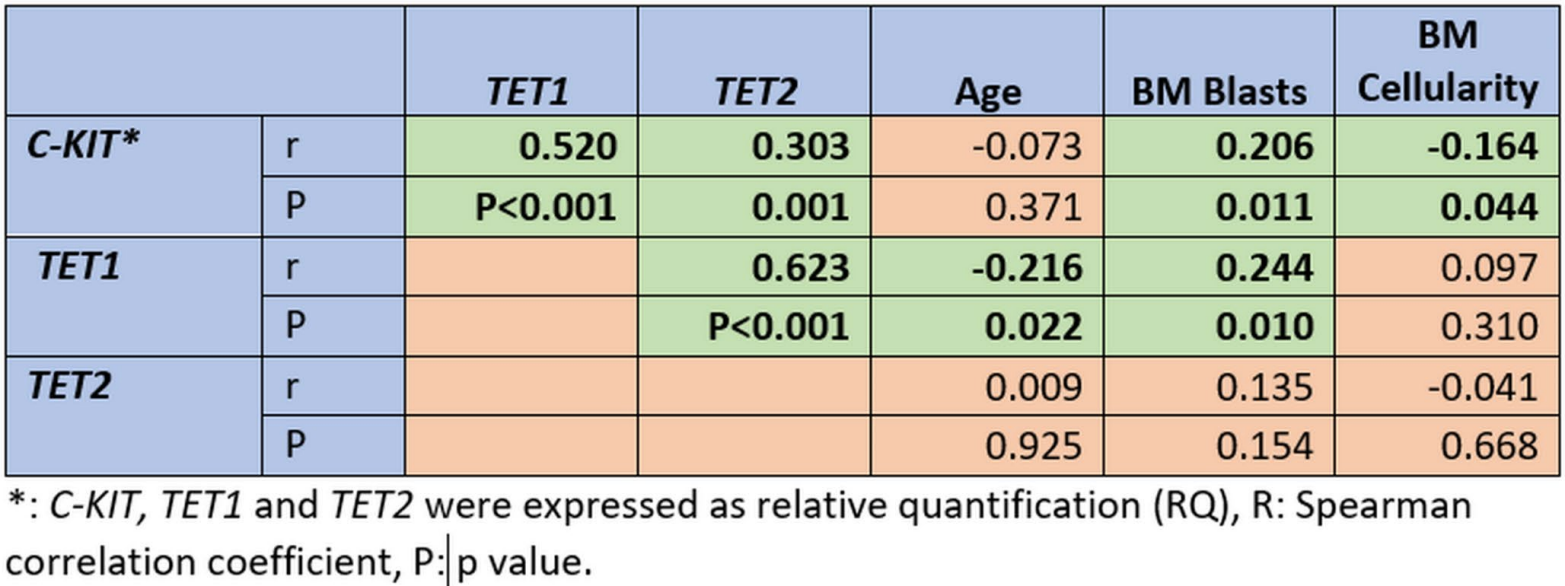
correlation between *TET1, TET2*, *CKIT*, blast percentage, patient age and BM cellularity in AML patients

There was a significant association between *c-Kit* overexpression and increased PB blast cell%, *FLT3-TKD* mutation, and disease relapse (P = 0.036, 0.004 and 0.019; respectively). However, *C-KIT* overexpression associated with cytogenetic of favorable prognosis and low-risk stratification (P = 0.036 and P = 0.042; respectively, Table 4). There was a significant correlation between the relative quantification (RQ) of *CKIT* expression and TET1 (r = 0.520 P < 0.001), TET2 (r = 0.303, P = 0.001), BM blasts% (r = 0.206, P = 0.011). while there was a weak inverse correlation with BM celluarity (r=-0.164, P = 0.044, Fig. 2).

**Table 4:** Association between *TET1* and *TET2* expression with the clinical features of the patients

		***TET1*** **expression**		**P value**	***TET2*** **expression**		**P value**
		**Low expression**	**Over** **expression**		**Low expression**	**Over** **expresion**	
Age (years)	median(range)	36 (1–64)	29 (1–62)	0.063	29 (1–64)	34 (1–64)	0.781
TLC	median(range)	17.9 (1-395)	30 (1-616)	0.044	16 (1-177)	31 (1-616)	0.036
Hb	median(range)	8 (4–14)	7.8 (4–12)	0.332	7.9 (4–14)	7.7 (4–13)	0.813
PLT	median(range)	34 (5-244)	33 (5-283)	0.751	33 (6-244)	24 (5-283)	0.631
PB. Blasts	median(range)	39 (0–90)	50 (0–98)	0.030	40 (0–90)	47 (0–98)	0.182
BM blasts	median(range)	65 (20–97)	71 (25–97)	0.026	65 (20–96)	71 (25–97)	0.306
Sex	male	32 (56.1%)	27 (49.1%)	0.570	29 (54.7%)	30 (51.7%)	0.849
	female	25 (43.9%)	28 (50.9%)		24 (45.3%)	28 (48.3%)	
BM Cellularity Initial	hypercellularity	46 (82.1%)	44 (80.0%)	0.352	41 (77.4%)	49 (86.0%)	0.483
	normocelluarity	10 (17.9%)	9 (16.4%)		11 (20.8%)	7 (12.3%)	
	hypocellularity	0 (0.0%)	2 (3.6%)		1 (1.9%)	1 (1.8%)	
FAB	M0	1 (1.8%)	1 (1.8%)	0.598	1 (1.9%)	1 (1.7%)	0.981
	M1	9 (15.8%)	12 (21.8%)		11 (20.8%)	9 (15.5%)	
	M2	18 (31.6%)	17 (30.9%)		16 (30.2%)	19 (32.8%)	
	M3	8 (14.0%)	5 (9.1%)		6 (11.3%)	7 (12.1%)	
	M4	18 (31.6%)	15 (27.3%)		15 (28.3%)	18 (31.0%)	
	M5	3 (5.3%)	2 (3.6%)		2 (3.8%)	3 (5.2%)	
	M7	0 (0.0%)	3 (5.5%)		2 (3.8%)	1 (1.7%)	
IPT	mono	2 (3.6%)	1 (1.9%)	0.309	2 (3.8%)	1 (1.8%)	0.822
	myelo	36 (64.3%)	33 (63.5%)		33 (63.5%)	35 (63.6%)	
	myelomono	18 (32.1%)	15 (28.8%)		15 (28.8%)	18 (32.7%)	
	megakaryoblastic	0 (0.0%)	3 (5.8%)		2 (3.8%)	1 (1.8%)	
FLT3-ITD	wild	46 (83.6%)	44 (80.0%)	0.805	41 (80.4%)	48 (82.8%)	0.807
	mutant	9 (16.4%)	11 (20.0%)		10 (19.6%)	10 (17.2%)	
FLT3-TKD	wild	19 (82.6%)	16 (66.7%)	0.318	16 (80.0%)	19 (70.4%)	0.517
	mutant	4 (17.4%)	8 (33.3%)		4 (20.0%)	8 (29.6%)	
NPM	wild	31 (70.5%)	38 (82.6%)	0.216	30 (71.4%)	38 (80.9%)	0.327
	mutant	13 (29.5%)	8 (17.4%)		12 (28.6%)	9 (19.1%)	
molecular translocations	negative	40 (70.2%)	40 (72.7%)	0.838	40 (75.5%)	39 (67.2%)	0.529
	t(8;21)	7 (12.3%)	8 (14.5%)		5 (9.4%)	10 (17.2%)	
	inv. 16	3 (5.3%)	3 (5.5%)		2 (3.8%)	4 (6.9%)	
	PML/RARa	7 (12.3%)	4 (7.3%)		6 (11.3%)	5 (8.6%)	
Cytogenetics	Favourable	28 (60.9%)	25 (49.0%)	0.308	26 (59.1%)	26 (50.0%)	0.416
	adverse	18 (39.1%)	26 (51.0%)		18 (40.9%)	26 (50.0%)	
genetic risk	HR	7 (12.3%)	11 (20.0%)	0.366	9 (17.0%)	9 (15.5%)	0.968
	IR	15 (26.3%)	17 (30.9%)		15 (28.3%)	16 (27.6%)	
	LR	35 (61.4%)	27 (49.1%)		29 (54.7%)	33 (56.9%)	
ELN classification	AR	5 (8.9%)	4 (7.1%)	0.832	4 (7.5%)	5 (8.6%)	0.564
	IR	35 (62.5%)	38 (67.9%)		37 (69.8%)	35 (60.3%)	
	FR	16 (28.6%)	14 (25.0%)		12 (22.6%)	18 (31.0%)	
Organomegaly	no	25 (59.5%)	25 (58.1%)	0.910	22 (55.0%)	28 (63.6%)	0.861
	hepatomegaly	4 (9.5%)	4 (9.3%)		4 (10.0%)	4 (9.1%)	
	splenomegaly	3 (7.1%)	5 (11.6%)		4 (10.0%)	4 (9.1%)	
	HSM	10 (23.8%)	9 (20.9%)		10 (25.0%)	8 (18.2%)	
LNs	no	21 (51.2%)	21 (44.7%)	0.669	18 (46.2%)	24 (50.0%)	0.830
	yes	20 (48.8%)	26 (55.3%)		21 (53.8%)	24 (50.0%)	
Relapse	no	29 (80.6%)	31 (83.8%)	0.768	30 (81.1%)	29 (82.9%)	0.845
	yes	7 (19.4%)	6 (16.2%)		7 (18.9%)	6 (17.1%)	
Early death before D28	no	41 (71.9%)	32 (58.2%)	0.165	35 (66.0%)	37 (63.8%)	0.844
	yes	16 (28.1%)	23 (41.8%)		18 (34.0%)	21 (36.2%)	
late death after d28	no	41 (71.9%)	44 (80.0%)	0.380	41 (77.4%)	44 (75.9%)	0.852
	yes	16 (28.1%)	11 (20.0%)		12 (22.6%)	14 (24.1%)	
Death	no	25 (43.9%)	20 (36.4%)	0.446	23 (43.4%)	22 (37.9%)	0.569
	yes	32 (56.1%)	35 (63.6%)		30 (56.6%)	36 (62.1%)	

AR: advanced risk, BM: bone marrow, FR: favourable risk, LR: low risk, HB: haemoglobin: HR: high risk, IPT: immunophenotyping, IR: intermediate risk, PB: peripheral blood, BM: bone marrow, PLT: platelets, TLC: total leukocyte count

### Association between *TET1* and *TET2* expression levels and the clinical features of the patients

*TET1* overexpression associated significantly with increased TLC count, PB blast%, and BM blast% (P = 0.044, 0.030, and 0.026; respectively, Table 4). While TET2 overexpression associated significantly with increased TLC count only in the recruited AML patients (P = 0.036, Table 5). However, there was no significant association between *TET1* and *TET2* expression levels and the other clinicopathological features assessed.

**Table 5:** Multivariate analysis for OS and DFS rates of the AML patients

	**Overall survival**			**Disease Free Survival**		
	**HR**	**SE**	**Sig.**	**HR**	**SE**	**Sig.**
**Univariate analysis**						
Age	1.142	0.209	0.526	2.662	0.532	0.065
Sex	1.225	0.206	0.327	0.589	0.580	0.362
HB	0.924	0.209	0.705	0.440	0.541	0.129
TLC	1.307	0.208	0.199	1.150	0.526	0.791
PLT	1.075	0.209	0.729	1.284	0.503	0.619
BM blast%	1.360	0.206	0.136	8.927	0.668	0.001
PB blast%	1.431	0.210	0.087	1.801	0.509	0.248
BM cellularity	0.688	0.246	0.129	1.687	0.757	0.490
Genetic risk	1.424	0.259	0.172	2.789	0.588	0.081
Cytogenetic	1.475	0.223	0.081	0.900	0.653	0.872
ELN stage	1.131	0.370	0.739	1.795	0.770	0.448
LNs involvement	2.064	0.246	0.003	0.942	0.558	0.914
IPT	0.998	0.237	0.257	0.860	0.543	0.782
Organomegaly	1.294	0.250	0.302	0.358	0.599	0.086
FAB1	1.078	0.218	0.731	1.401	0.509	0.508
*FLT3-ITD* mutation	0.801	0.280	0.427	3.770	0.507	0.009
*FLT3-TKD* mutation	0.703	0.410	0.390	2.843	0.920	0.256
*NPM* mutation	0.480	0.321	0.022	0.481	1.049	0.485
*c-KIT*	0.811	0.212	0.321	2.976	0.584	0.062
*TET1*	1.324	0.247	0.257	1.212	0.675	0.776
*TET2*	1.242	0.249	0.385	1.578	0.612	0.456
**Multivariate analysis**						
LNs involvement	2.200	0.280	0.005			
*NPM*	0.567	0.354	0.110			
BM Blast%				7.768	0.671	0.002
*FLT3-ITD*				2.989	0.511	0.032

BM: bone marrow, HB: haemoglobin, HR: hazard ratio, IPT: immunophenotyping, PB: peripheral blood, BM: bone marrow, PLT: platelets, TLC: total leukocyte count

*TET1* correlated significantly with *TET2* expression (r = 0.623, P < 0.001), BM blasts% (r = 0.244, P = 0.010), and patients’ age (r=-0.216, P = 0.022, Fig. 2).

### Impact of *c-Kit, TET1* and *TET2* expression levels on the DFS and OS rates of the AML patients

**Fig. 3:**
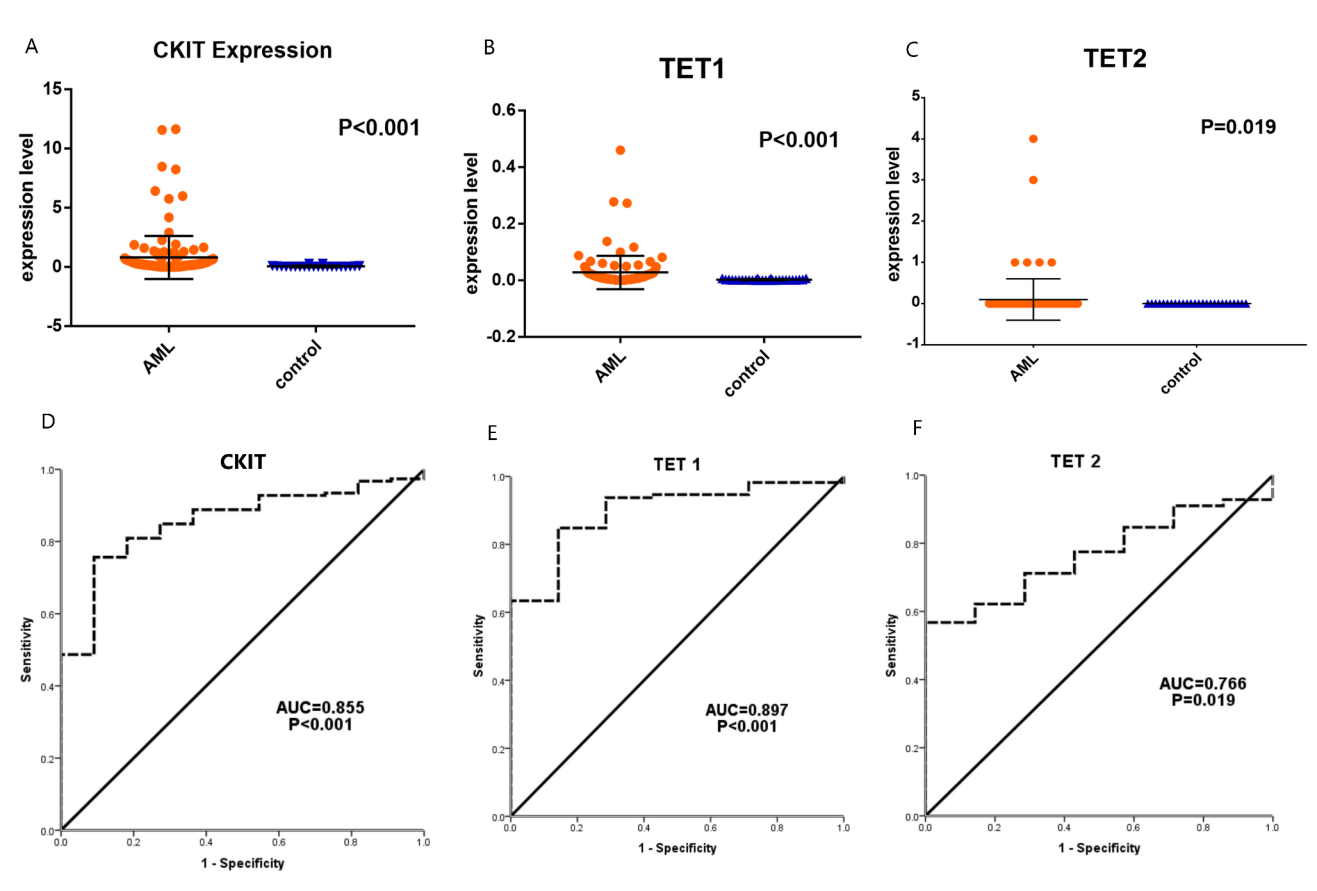
Association of (a) *C-KIT*, (b) *TET1*, (c) *TET2* and (d) *C-KIT + TET1* expression with the disease free survival of the patients. Association of (e) *C-KIT*, (f) *TET1*, (g) *TET2* and (h) *C-KIT + TET1* expression with the overall survival rates of the AML patients

The AML patients who had *c-KIT* overexpression showed a significantly lower DFS rate compared to those with *c-KIT* low-expression, as the mean DFS in *c-KIT* overexpressors was 18.183 months compared to 24.212 months in those with *c-KIT* low-expression (P = 0.05). However, there was no significant association between *c-KIT* expression and OS rates of the patients. Similarly, there was no significant impact of *TET1* nor *TET2* expression levels and OS or DFS rates of the recruited AML patients (Fig. 3).

### Univariate and multivariate analysis of the patients

The univariate analysis showed that patients with lymph nodes (LN) involvement [HR = 2.064, P = 0.003] and/or mutant NPM [HR = 0.480, P = 0.022] had significantly lower OS rates. While patients with increased BM blast% [HR = 0.668, P = 0.001], and/or those with *FLT3-ITD* mutation [HR = 0.507, P = 0.009] showed a significant lower DFS rates.

On the other hand, multivariate analysis showed that LN involvement [HR = 2.200, P = 0.005] is an independent risk factor for the shorter OS rate of AML patients. While increased BM blast% [HR = 7.768, P = 0.002], and *FLT3-ITD* mutation [HR = 2.989, P = 0.032] are independent risk factors for shorter DSF rate of the patients (Table 5).

### The prognostic accuracy of *CKIT, TET1* and *TET2* for AML patients

The prognostic accuracy of *CKIT, TET1* and *TET2* was assessed in relation to the established prognostic factors including patient age, ELN risk stage, and BM blasts %. The data showed that the AUC for patient age, ELN risk stage, and BM blasts % for predicting relapse of the AML patients was 0.646 (P = 0.049), and when adding *CKIT*, the AUC increased to 0.740 (P = 0.001). While when using patient age, ELN risk stage, BM blasts %, *CKIT, TET1* and *TET2*, the AUC increased to 0.743 (P = 0.005). Regarding the survival of the patients, patient age, ELN risk stage, and BM blasts % achieved (AUC: 0.623, P = 0.012), whlie when adding *CKIT, TET1* and *TET2*, the AUC become 0.622 (P = 0.031, Table 6).

**Table 6:** The prognostic accuracy of *CKIT, TET1* and *TET2* for AML patients

	**Relapse**					**Survival**				
	**AUC**	**Std. Error**	**P value**	**95% CI**		**AUC**	**Std. Error**	**P value**	**95% CI**	
patient age + ELN + BM blasts %	0.646	0.064	0.049	0.522	0.771	0.623	0.046	0.012	0.533	0.713
patient age + ELN + BM blasts %+ CKIT	0.740	0.060	0.001	0.622	0.858	0.612	0.046	0.023	0.522	0.701
patient age + ELN + BM blasts %+ TET1	0.654	0.066	0.066	0.524	0.784	0.619	0.054	0.034	0.514	0.724
patient age + ELN + BM blasts %+ TET2	0.666	0.063	0.054	0.542	0.791	0.616	0.054	0.037	0.510	0.723
patient age + ELN + BM blasts %+ CKIT + TET1 + TET2	0.743	0.066	0.005	0.613	0.872	0.622	0.053	0.031	0.517	0.727

AUC: area under curve, CI: confidence interval

## Discussion

AML is the most common hematological malignancy worldwide. Though the advancement of the diagnostic and therapeutic modalities, still, there is an increased incidence of disease relapse and lower survival rates in AML patients. DNA methylation plays an important role in AML carcinogenesis, however, few studies had investigated the diagnostic and prognostic roles of *TETs* expression in AML [29]. The current study aimed at investigating the role of *C-KIT, TET1*, and *TET2* expression levels and their clinical significance in AML patients.

The present data showed that *C-KIT* was significantly up-regulated in AML cases compared to the control group with a diagnostic power of 48.7% sensitivity and 100% specificity. These data are in agreement with other studies reported up-regulation of *C-KIT* in AML patients compared to the control group [37–40]. Abrams et al., found that C-KIT overexpression was recognized in many cancers including gastrointestinal stromal tumors, small cell lung cancer, melanoma, non–small cell lung cancer, and AML, which raise the attention for using an anti-C-KIT antibody as a treatment protocol in these cancers [37]. Moreover, *C-KIT* up-regulation was a bad prognostic factor for AML patients, as its upregulation was significantly associated with a shorter DFS rate. This came in concordance with Advani et al. who found that increased intensity of *C-KIT* using the mean fluorescence index correlates with a decreased progression-free survival (PFS) and OS rates in AML patients [38]. Also, Gao et al. found that PFS was significantly shorter in patients with high *C-KIT* expression [39].

In the current study, *C-KIT* overexpression associated significantly with increased peripheral blood blasts and *FLT3-TKD* mutation. These data are in agreement with Hoehn et al. [40], who concluded that *CD117(C-KIT)* expression is a sensitive marker for FLT3 mutation in AML. However, Woźniak and Kopeć-Szlezak [41] found a negative correlation between the level of *C-Kit* expression and the peripheral blood blasts. This discrepancy may be due to the different methods used for the detection of *C-Kit* expression, as they used a relative fluorescence intensity using immunophenotypic analysis [40]. In addition, *C-KIT* overexpression showed a significant association with disease relapse in AML patients. These results are similar to that observed by Gao et al. [39], who found that patients with *C-KIT* overexpression showed poor disease outcomes. Moreover, our data demonstrated that lower expression of *C-KIT* was found in patients with low-risk prognostic factors, and its overexpression associated significantly with karyotypes of favorable prognosis [AML with t(8;21) and (inv-16)]. These data are in concordance with that reported by Gao et al. and Auewarakul et al., who found a significant association between *C-KIT* overexpression and the presence of AML with t(8;21) and/or inv-16 [39–42].

The current study showed also that *TET1*and *TET2* were significantly upregulated in the BM samples of AML patients compared to the control group. In addition, *TET1, TET2*, and *C-KIT* could possibly add a diagnostic value for AML patients based upon the results obtained from the ROC curve analysis. It showed that *TET2* achieved the highest sensitivity (63.4%) compared to *TET2* (56.8%) and *C-KIT* (48.7%) expression at a specificity of 100%, while when combining the three markers together, the sensitivity was 77.5%. However, it reached the highest sensitivity (78.6%) and specificity (100%) when combining the expression of both *c-KIT + TET1* together for the diagnosis of AML. Actually, the expression levels of *TET1* and *TET2* mRNA in AML patients are still a debatable issue. Zhang et al. (2020), found that *TET1* was down-regulated, while *TET2* was up-regulated in AML patients [29]. Similarly, Cheng et al. [43], observed a significant upregulation of *TET2* in AML patients. However, Zhang et al. (2018) found that TET2 mRNA level was significantly down-regulated in AML patients compared with control subjects [16]. This discrepancy in the results may be due to the differeneces in the detection assay or the housekeeeping gene used. As well as the different cells used for the assessment either using unsorted whole BM cells or using sorted BM blasts, in addition to the different sample size of the patients.

Regarding the prognostic value of *TET1* and *TET2* in AML patients, our results could not confirm that *TET1* and *TET2* overexpression were a bad prognostic factor for AML, as we were not able to find a significant association between *TET1* or *TET2* expression and survival rates (DFS and OS) of the patients. This came in agreement with Zhang et al. [29], who found that there is no significant associations between *TET1* or *TET2* expression and the survival rates of the assessed patients. However, Wang et al. [17] proposed that high expression of *TET1* was significantly associated with poor prognosis of the patients in the form of shorter OS and DFS rates in cytogenetically normal AML patients. Though, this was not applied to patients with high expression of *TET2.* This difference in the results could be attributed to the impact of using unsorted bone marrow cells other than using blast cells, in addition to that some patients included in the current study had AML with cytogenetic abnormalities.

The present results showed that TET1 overexpression associated significantly with high TLC together with a high number of PB blasts and BM blasts, while it had no significant association with age, FLT3/TKD mutation, molecular translocations nor any of the FAB subtypes. These data are consistent with Wang et al. [17], who couldn’t confirm any significant association between TET1 overexpression and patients’ ages or FLT3 mutation, however, they found that the cytogenetically normal AML patients with high TET1 expression showed a higher frequency of NPM1 mutations and FAB M0/1 morphology, while there was no significant association between TET1 expression and TLC, PB nor BM blasts. This controversy may be due to the impact of using unsorted bone marrow cells, or using different detection methods (e.g. different housekeeping gene). In addition to the difference in the number and type of the patients, as they recriuted a larger number of patients who were cytogenetically normal AML.

As regards TET2, its overexpression associated significantly with a high total leukocyte count. However, there was no significant association with age, sex, hemoglobin level, platelet count, percentage of PB blasts, BM blasts, cytogenetic abnormality, FLT3, or NPM mutations.

These data are comparable to that observed by Zhang et al. [16], who concluded similar results except that patients with low TET2 expression showed a high frequency of NPM1 mutations. Also, they found a significant association between TET2 expression and FAB subtypes of leukemia. This controversy could be attributed to the large number of patients who had NPM1 mutation included in their study, compared to the small number available for the present study [16].

Multivariate analysis showed that LN involvement is an independent risk factor for the shorter OS rate of AML patients. While increased BM blast% and *FLT3-ITD* mutation are independent risk factors for shorter DSF rate of the patients. However, when correlating the prognostic accuracy of the assessed genes to the established prognostic factors including patient age, ELN risk stage, and BM blasts %, it revelaed that adding *CKIT, TET1, or TET2* could impove the prognosic power for prediciting relapse in AML patients. Moreover, when combining patient age, ELN risk stage, BM blasts %, *CKIT, TET1* and *TET2* together, the prognostic power became greater. On the other hand, adding *CKIT, TET1* and *TET2* had no significant impact on predicting patients’ survival. These data are in agreement with Liu et al. [44], who reported that TET2 mutations had an adverse impact on the prognosis of AML patients. Also, Wang et al. [17] proposed that TET1 was a reliable prognostic factor for patients with AML.

In conclusion, C-KIT, TET1, and TET2 were upregulated in newly diagnosed AML patients compared to the control subjects. All of them associated with specific phenotypes of AML indicating that these biological biomarkers may be helpful in providing new insights in understanding the pathogenesis of AML. Additionally, *C-KIT, TET1*, and *TET2* could be used as potential useful markers for the diagnosis of AML especially when combined together as *c-KIT + TET1 + TET2* achieved a sensitivity of 77.5%, and specificity of 100%, while *c-KIT + TET1* achieved a sensitivity of 78.6%, and a specificity of 100%. Moreover, multivariate logistic regression analysis showed that *CKIT* and *TET1* could be considered as possible independent predictors for AML diagnosis. Also, *C-KIT, TET1 and TET2* proved to be a negative prognostic biomarker for relapse in AML patients. However, these results should be validated on a larger number of samples with more categorization of patients according to their age, risk, and leukemia subtypes.
